# The impairment of the deep vascular complex in prolonged type 2 diabetes patients without clinical diabetic retinopathy

**DOI:** 10.1371/journal.pone.0269182

**Published:** 2022-06-03

**Authors:** Tae-Yeon Kim, Yong-Yeon Song, Il-Jung, Yong-Jin Na, Young-Hoon Lee, Jung-Yeul Kim, Min-Woo Lee

**Affiliations:** 1 Department of Ophthalmology, Konyang University College of Medicine, Daejeon, Republic of Korea; 2 1.0 Eye Clinic, Daejeon, Republic of Korea; University of Utah (Salt Lake City), UNITED STATES

## Abstract

**Purpose:**

To identify the effects of prolonged type 2 diabetes (T2DM) on the retinal microvasculature of each retinal capillary plexus in patients without clinical diabetic retinopathy (DR).

**Methods:**

Subjects were divided into three groups: the control group (98 eyes), patients with T2DM < 10 years (DM group 1, 84 eyes), and patients with T2DM ≥ 10 years (DM group 2, 55 eyes). The vessel densities (VD) of the superficial and deep capillary plexus (SCP and DCP) were compared. Linear regression analyses were performed to identify factors associated with the VD.

**Results:**

The mean VDs of the SCP in the control group, DM group 1, and DM group 2 were 35.9 ± 4.2, 34.9 ± 3.9, and 34.6 ± 5.1, respectively (*P =* 0.042). The mean VDs of the DCP in the three groups were 36.1 ± 3.1, 35.9 ± 3.0, and 34.0 ± 3.3, respectively (*P <* 0.001). In multivariate analyses, the BCVA was a significant factor associated with both the superficial VD (B = −7.10, *P* = 0.019) and deep VD (B = −5.70, *P* = 0.039). Hypertension (B = −1.22, *P* = 0.021) and DM duration (B = −0.20, *P* < 0.001) were significant factors associated with deep VD.

**Conclusions:**

T2DM patients without DR showed decreased VD in the SCP and DCP. The microvascular impairment of the DCP in patients with T2DM ≥ 10 years was in particular, more severe. Additionally, ischemia caused by hypertension and accumulated impairment of microvasculature due to prolonged T2DM would affect the DCP.

## Introduction

Diabetic retinopathy (DR), the most common microvascular complication of diabetes, remains the leading cause of blindness among working age individuals in the most developed countries [1, 2]. DR is commonly accompanied by microvascular dysfunction characterized by microaneurysms, hemorrhages, lipid exudates, macular edema, capillary occlusion, cotton wool spots, and finally neovascularization [3]. Meanwhile, recent studies reported that retinal damage can occur even for patients with type 2 DM (T2DM) showing no signs of clinical DR [4–6]. Lim et al. observed faster reduction rates of the peripapillary retinal nerve fiber layer (pRNFL) in patients with T2DM without DR than those of the control group [4]. Previous studies have also found that retinal damage caused by T2DM could result in thinning of the ganglion cell–inner plexiform layer (GCIPL) and pRNFL in T2DM patients without clinical DR [5, 6]. Such inner retinal damage is known as the diabetic neurodegeneration, which is representative retinal damage anteceding clinical DR [7].

Additionally, with the development of a device capable of observing retinal microvasculature, studies on the retinal microvasculature of T2DM patients without DR have been actively conducted. Optical coherence tomography angiography (OCTA), a noninvasive imaging modality for visualizing the microvasculature of the retina and choroid, allows depth-resolved imaging of the retinal vasculature [8]. Many studies have reported the microvasculature in patients with T2DM without DR using OCTA [9, 10]. Cao et al. found a decrease in the parafoveal vessel density (VD) of both the superficial and deep capillary plexus (SCP and DCP) in the diabetic eyes without clinical DR [9]. Lee et al. reported thinner RNFL and lower superficial peripapillary VD and perfusion density in patients with T2DM without clinical DR compared with normal controls. Such damage was found to be more severe in patients with T2DM ≥ 10 years [10]. However, few studies have reported the damage of each retinal capillary plexus in patients with prolonged T2DM.

The purpose of this study was to identify the effects of prolonged T2DM on the retinal microvasculature of each retinal capillary plexus by comparing the VD of the SCP and DCP in patients with T2DM for < 10 years and patients with T2DM for ≥ 10 years. The factors associated with the microvasculature of each capillary plexus in patients with T2DM were also investigated.

## Materials and methods

### Patients

This retrospective, cross-sectional study adhered to the tenets of the Declaration of Helsinki and was approved by the Institutional Review Board of Konyang University Hospital, Daejeon, the Republic of Korea (IRB No. 2021-05-014). The requirement of informed consent from patients was waived off because of the retrospective nature of the study. We reviewed the charts of patients with T2DM who visited the retina clinic of Konyang University Hospital for DR checkups from March 2017 to December 2020. All patients were followed-up in the Department of Internal Medicine of Konyang University Hospital and the diagnosis of T2DM (fasting plasma glucose of ≥ 126 mg/dL or 2-hour plasma glucose of ≥ 200 mg/dL or hemoglobin A1c of ≥ 6.5%) and hypertension (clinical blood pressure, ≥ 140/90 mmHg; home blood pressure, ≥ 135/85 mmHg) was made according to the criteria of the American Diabetes Association and the Korean hypertension treatment guideline [11, 12]. We enrolled the control group diagnosed with unilateral epiretinal membrane, macular hole, or intraocular lens dislocation; all of the fellow eyes without any ophthalmic disease. Each patient underwent a complete ophthalmic examination, which included the best-corrected visual acuity (BCVA) and intraocular pressure (IOP) using non-contact tonometry, refraction, and axial length using an IOL Master 500 (Carl Zeiss, Jena, Germany). We used a Snellen visual acuity chart to measure the BCVA, which was converted to the minimum angle of resolution (logMAR). We divided the subjects among three groups: control, patients with T2DM < 10 years (DM group 1), and patients with T2DM ≥ 10 years (DM group 2), which was referring to previous studies and maintaining a sufficient number of patients in each group to allow for statistical analysis in this study [13, 14]. Exclusion criteria were as follows: a history of any systemic disease other than T2DM and hypertension; any ophthalmic disease such as glaucoma, retinal diseases, or neuro-ophthalmic diseases; axial length ≥ 26.0 mm; any prior intraocular surgery except cataract extraction; a BCVA < 0.7; an IOP ≥ 21 mmHg. We also excluded patients with clinical evidence of DR, such as retinal hemorrhage or microaneurysms. One eye was randomly selected from patients who met the inclusion criteria.

### Optical coherence tomography (OCT) measurements

An experienced examiner obtained the measurements using SD-OCT (Spectralis; Heidelberg Engineering, Heidelberg, Germany). Foveal measurements obtained using SD-OCT were analyzed using the built-in Spectralis mapping software (Heidelberg Eye Explorer, version 6.9a). We used the retinal thickness map analyses to display the numerical averages of the measurements for each of the parafoveal Early Treatment Diabetic Retinopathy Study (ETDRS) subfields. The inner (subfovea) and intermediate rings (parafovea) with diameters of 1 mm and 3 mm, respectively, were analyzed. The central macular thickness and average thicknesses of the parafoveal areas were used for the analyses: the inner superior, inner inferior, inner nasal, and inner temporal. Automated retinal layer segmentation was conducted, and the thickness of each inner retinal layer of the parafoveal area was determined using automated Spectralis segmentation software. The thicknesses of the full retina, retinal nerve fiber layer (NFL), ganglion cell layer (GCL), and inner plexiform layer (IPL) were estimated.

### VD measurement using OCTA

OCTA was performed using a Spectralis OCT2 device (Heidelberg Engineering, Heidelberg, Germany). The Spectralis OCT2 instrument is capable of 70,000 A-scans per second using a light source centered at 870 nm, with the axial and transverse resolutions of 3.9 μm and 6 μm in the tissue, respectively. En face images of the SCP, defined as the layer originating from the internal limiting membrane to the inner plexiform layer, and the DCP, defined as the layer starting from the outer border of the inner plexiform layer to the outer plexiform layer, were visualized automatically by segmenting two separate slabs defined by arbitrary segmentation lines, which are created by the device software. In this study, a 3.0 × 3.0 mm OCTA scan centered around the fovea was acquired to analyze the posterior pole area around the macula. The VD was calculated using ImageJ software (National Institutes of Health, Bethesda, MD, USA) [15]. In particular, 8-bit images were split into red, green, and blue channels, with the red channel used as the reference. The adjusted threshold tool was applied with default settings, and the dark background option was selected. This tool automatically sets the lower and upper threshold values (arbitrarily chosen as 110 and 255, respectively, for every image) and segmented grayscale images into features of interest and background. The foveal avascular zone (FAZ), defined as the avascular area in the center of the fovea, was manually outlined by two graders (T.Y.K and M.W.L). A built-in program allowing the measurement of the outlined areas was deployed to estimate the area. The mean values of the two measurements were used for the analysis. Images with loss of fixation, segmentation errors, and motion artifacts were excluded. The OCTA quality is literally the quality of OCTA that the device automatically measured in consideration of the image quality and clarity of the OCTA device. The OCTA quality score ranged from 0 (no signal) to 40 (excellent quality). The quality bar turned red when the score was ≤ 15, indicating poor scan quality; the scan quality was considered marginal when the score was between 15 and 25; in case the score was ≥ 25, the scan quality was considered good. We excluded the OCTA images with an OCTA quality of < 20.

### Statistical analysis

One-way ANOVA with post hoc Bonferroni correction and the chi-square test was used to compare the demographic characteristics and ocular parameters. Univariate and multivariate linear regression analyses were performed to identify factors associated with superficial and deep macular VD in patients with T2DM. Statistical analyses were performed using SPSS statistical software for Windows (version 18.0; IBM, Armonk, New York, USA).

## Results

### Demographics

A total of 237 eyes were enrolled for the study: 98 in the control group, 84 in the DM group 1, and 54 in the DM group 2. The mean age of each group was 56.1 ± 11.6 years, 55.8 ± 13.9 years, and 60.6 ± 10.7 years, respectively (*P* = 0.063, Table 1).

**Table 1:** Demographics and clinical characteristics.

	Normal controls (n = 98)	DM group 1 (n = 84)	DM group 2 (n = 55)	P value
Age (years)	56.1 ± 11.6	55.8 ± 13.9	60.6 ± 10.7	0.063
Sex (male, %)	43 (43.8)	52 (61.9)	29 (52.7)	0.052
Laterality (right, %)	45 (45.9)	50 (59.5)	28 (50.9)	0.130
BCVA (logMAR)	0.032 ± 0.052	0.050 ± 0.133	0.062 ± 0.070	**0.018** (0.147*, **0.020**^**†**^, 0.861^‡^)
SE (diopters)	-0.89 ± 2.14	-0.06 ± 1.51	-0.21 ± 1.36	0.060
IOP (mmHg)	13.2 ± 3.0	13.8 ± 3.3	13.2 ± 3.3	0.356
Axial length (mm)	24.1 ± 1.1	23.8 ± 1.1	23.9 ± 0.8	0.440
Hypertension (%)	46 (46.9)	41 (48.8)	35 (63.6)	0.102
DM duration (years)	0	3.2 ± 2.7	14.9 ± 5.9	**< 0.001**
HbA1C (%)	N/A	7.1 ± 1.9	7.0 ± 1.0	0.794
CMT (μm)	268.8 ± 23.2	264.4 ± 22.6	262.0 ± 22.2	0.173
Parafoveal NFL thickness (μm)	23.0 ± 2.5	22.5 ± 2.8	22.1 ± 3.4	0.104
Parafoveal GCL thickness (μm)	50.1 ± 5.0	48.0 ± 5.8	47.7 ± 6.9	**0.019** (0.024*, 0.010^**†**^, 0.965^‡^)
Parafoveal IPL thickness (μm)	40.8 ± 3.4	39.6 ± 3.7	39.3 ± 4.1	**0.023** (0.026*, 0.016^†^, 0.925^‡^)
OCTA quality (mean ± SD dB)	33.6 ± 2.6	33.2 ± 2.3	33.2 ± 2.1	0.150

SD, standard deviation; BCVA, best-corrected visual acuity; SE, spherical equivalent; IOP, intraocular pressure; DM, diabetes; CMT, central macular thickness; NFL, nerve fiber layer; GCL, ganglion cell layer; IPL, inner plexiform layer; DM group 1 = patients with type 2 diabetes < 10 years, DM group 2 = patients with type 2 diabetes ≥ 10 years.

All values are expressed as mean ± standard deviation.

Values in boldface (P < 0.05) are statistically significant.

*Control vs. DM group 1,

^†^control vs. DM group 2,

^‡^DM group 1 vs. DM group 2.

Sex, spherical equivalent, IOP, and axial length were not significantly different among the groups. The mean BCVA was 0.032 ± 0.052, 0.050 ± 0.133, and 0.062 ± 0.070, respectively, which were significantly different among the groups (*P* = 0.018) (post hoc: control group vs. DM group 1, *P* = 0.147; control group vs. DM group 2, *P* = 0.020; DM group 1 vs. DM group 2, *P* = 0.861). The HbA1c levels of the DM group 1 and DM group 2 were 7.1 ± 1.9% and 7.0 ± 1.0%, respectively (*P* = 0.794). The central macular thickness (CMT) and parafoveal nerve fiber layer thickness were not significantly different among the groups. However, the thickness of the parafoveal ganglion cell layer and parafoveal inner plexiform layer were significantly different among the groups (*P* = 0.018 and *P* = 0.023 respectively). Post hoc of the ganglion cell layer was: control group vs. DM group 1, *P* = 0.024; control group vs. DM group 2, *P* = 0.010; DM group 1 vs. DM group 2, *P* = 0.965 and the same for the inner plexiform layer was: control group vs. DM group 1, *P* = 0.026; control group vs. DM group 2, *P* = 0.016; DM group 1 vs. DM group 2, *P* = 0.925.

### The OCTA parameters in each group

The mean VDs of the SCP in the control group, DM group 1, and DM group 2 were 35.9 ± 4.2 μm, 34.9 ± 3.9 μm, and 34.6 ± 5.1 μm, respectively, and they were significantly different (*P =* 0.042) (Table 2) (post hoc: control group vs. DM group 1, P = 0.105; control group vs. DM group 2, P = 0.052; DM group 1 vs. DM group 2, P = 0.920).

**Table 2:** Optical coherence tomography angiography parameters in each group.

	Control	DM group 1	DM group 2	P-value
Vessel density (%)				
SCP	35.9 ± 4.2	34.9 ± 3.9	34.6 ± 5.1	**0.042**
DCP	36.1 ± 3.1	35.9 ± 3.0	34.0 ± 3.3	**< 0.001**
Choriocapillaris	41.3 ± 3.0	41.2 ± 2.9	41.0 ± 3.1	0.310
FAZ area (mm^2^)	0.23 ± 0.10	0.33 ± 0.12	0.48 ± 0.21	**< 0.001**

SCP, superficial capillary plexus; DCP, deep capillary plexus; FAZ, foveal avascular zone.

DM group 1 = patients with type 2 diabetes < 10 years, DM group 2 = patients with type 2 diabetes ≥ 10 years.

Values in boldface (P < 0.05) are statistically significant. All values are expressed as the mean ± standard deviation (μm).

**Fig 1:**
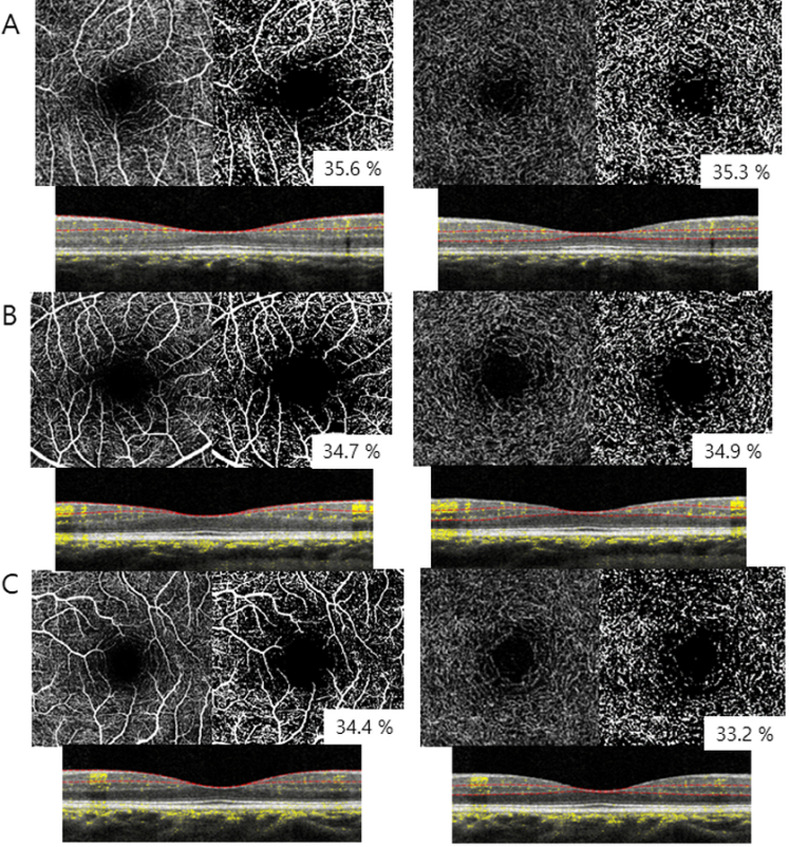
Representative original optical coherence tomography angiography images, images after conversion by ImageJ, and B-scan images of superficial capillary plexus and deep capillary plexus in control (A), DM group 1 (B), and DM group 2 (C).

The mean VD of DCP in the control group, DM group 1, and DM group 2 were 36.1 ± 3.1 μm, 35.9 ± 3.0 μm, and 34.0 ± 3.3 μm, respectively, and they also showed a significant difference among groups (*P <* 0.001) (post hoc: control group vs. DM group 1, *P* = 0.950; control group vs. DM group 2, *P* < 0.001; DM group 1 vs. DM group 2, *P* < 0.001). The mean VD of the choriocapillaris was not significantly different (*P* = 0.310) (Fig 1).

The FAZ areas in the control group, DM group 1, and DM group 2 were 0.23 ± 0.10 μm, 0.33 ± 0.12 μm, and 0.48 ± 0.21 μm, respectively. In the post hoc analyses, FAZ areas were significantly different between the control group and DM group 2 (*P* < 0.001), and between DM group 1 and DM group 2 (*P* < 0.001).

### Factors associated with the VD of the SCP in patients with T2DM

In univariate analyses, factors including the age (B = −0.07, *P* = 0.020), BCVA (B = −12.31, *P* < 0.001), hypertension (B = −1.73, *P* = 0.033), OCTA quality (B = 1.05, *P* < 0.001), CMT (B = 0.10, *P <* 0.001), parafoveal GCL thickness (B = 0.20, *P =* 0.001), and parafoveal IPL thickness (B = 0.35, *P =* 0.001) were significantly associated with the VD of the SCP in patients with T2DM (Table 3). In multivariate analyses, the BCVA (B = −7.10, *P* = 0.019), OCTA quality (B = 1.01, *P <* 0.001), and parafoveal GCL thickness (B = 0.16, *P <* 0.001) showed significant results.

**Table 3:** Univariate and multivariate linear regression analyses determining factors associated with vessel density of superficial capillary plexus in patients with type 2 diabetes.

	Univariate		Multivariate	
	B (95% CI)	P values	B (95% CI)	P values
Age	-0.07 (-0.15 to -0.02)	**0.020**	-0.02 (-0.07 to 0.05)	0.610
Sex	0.50 (-1.22 to 2.13)	0.620		
Laterality	-0.23 (-2.10 to 1.40)	0.730		
BCVA	-12.31 (-19.10 to -5.01)	**< 0.001**	-7.10 (-11.94 to -2.91)	**0.019**
SE	-0.11 (-0.64 to 0.47)	0.801		
IOP	-0.02 (-0.41 to 0.37)	0.910		
Axial length	-0.04 (-1.40 to 1.31)	0.955		
Hypertension	-1.73 (-3.41 to -0.07)	**0.033**	-0.91 (-2.07 to 0.10)	0.093
DM duration	-0.10 (-0.23 to 0.05)	0.310		
HbA1C	-0.04 (-0.55 to 0.40)	0.931		
OCTA quality	1.05 (0.88 to 1.31)	**< 0.001**	1.01 (0.72 to 1.30)	**< 0.001**
CMT	0.10 (0.04 to 0.16)	**< 0.001**	0.06 (-0.03 to 0.10)	0.425
Parafoveal NFL	0.18 (-0.10 to 0.49)	0.361		
Parafoveal GCL	0.20 (0.10 to 0.30)	**0.001**	0.16 (0.06 to 0.26)	**0.001**
Parafoveal IPL	0.35 (0.14 to 0.52)	**0.001**	-0.06 (-0.62 to 0.49)	0.811

BCVA = best-corrected visual acuity; SE = spherical equivalent; IOP = intraocular pressure; DM = diabetes; CMT = central macular thickness; NFL = nerve fiber layer; GCL = ganglion cell layer, IPL = inner plexiform layer.

Values in boldface (P < 0.05) are statistically significant.

### Factors associated with the VD of the DCP in patients with T2DM

**Fig 2:**
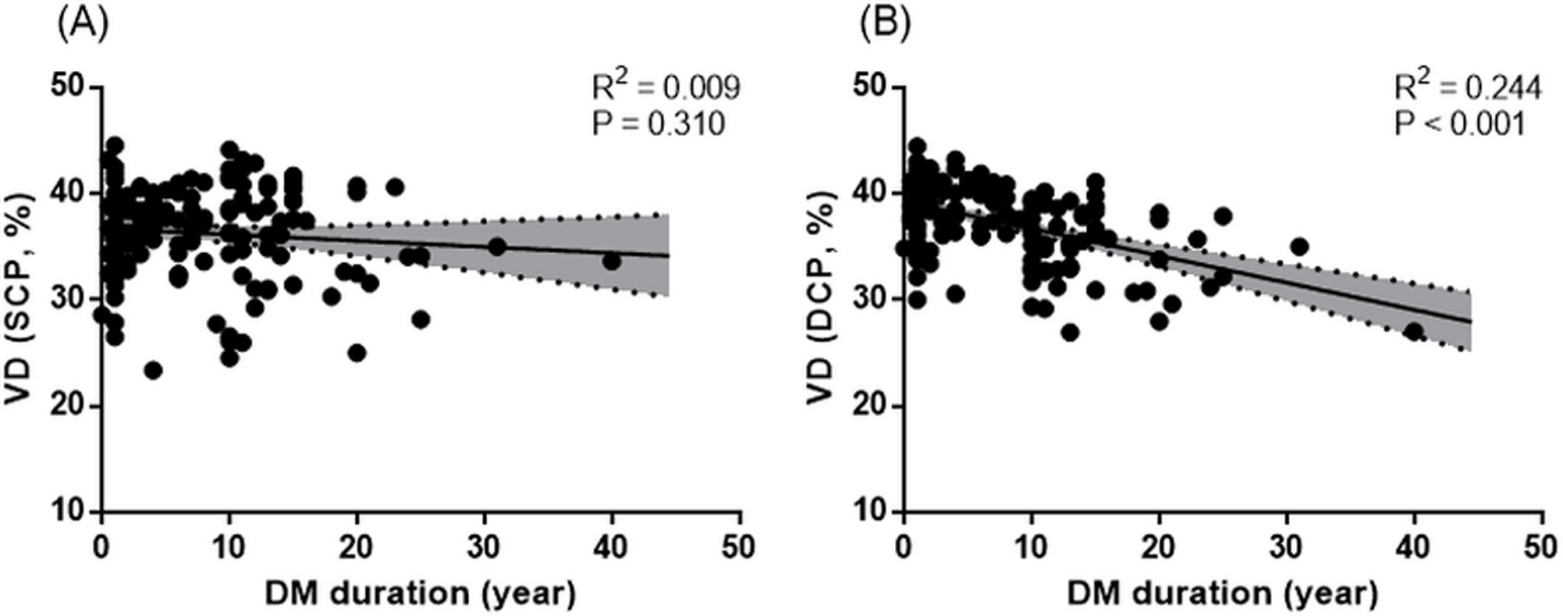
Scatterplots and linear regression analyses between the duration of T2DM and superficial vessel density (VD) (R^2^ = 0.009, p = 0.310) (A) and deep VD (R^2^ = 0.244, p<0.001) (B) in patients with T2DM.

In univariate analyses, the factors including the age (B = −0.07, *P =* 0.015), BCVA (B = −7.52, *P =* 0.010), hypertension (B = −1.90, *P <* 0.001), DM duration (B = −0.25, *P* < 0.001), and OCTA quality (B = 0.91, *P* < 0.001) were significantly associated with the VD of the DCP in patients with T2DM (Table 4). In multivariate analyses, the BCVA (B = −5.70, *P* = 0.039), hypertension (B = −1.22, *P* = 0.021), DM duration (B = −0.20, *P* < 0.001), and OCTA quality (B = 0.80, *P <* 0.001) showed significant results (Fig 2).

**Table 4:** Univariate and multivariate linear regression analyses determining factors associated with vessel density of deep capillary plexus in patients with type 2 diabetes.

	Univariate		Multivariate	
	B (95% CI)	P values	B (95% CI)	P values
Age	-0.07 (-0.12 to -0.02)	**0.015**	-0.01 (-0.06 to 0.05)	0.702
Sex	-0.20 (-1.23 to 0.90)	0.750		
Laterality	-1.12 (-3.40 to 1.22)	0.201		
BCVA	-7.52 (-13.46 to -2.19)	**0.010**	-5.70 (-10.30 to -1.13)	**0.039**
SE	0.07 (-0.40 to 0.51)	0.861		
IOP	0.17 (-0.05 to 0.31)	0.240		
Axial length	0.02 (-1.01 to 1.04)	0.940		
Hypertension	-1.90 (-2.81 to -1.02)	**< 0.001**	-1.22 (-2.24 to -2.20)	**0.021**
DM duration	-0.25 (-0.33 to -0.16)	**< 0.001**	-0.20 (-0.27 to -0.13)	**< 0.001**
HbA1C	0.03 (-0.36 to 0.42)	0.896		
OCTA quality	0.91 (0.70 to 1.16)	**< 0.001**	0.80 (0.51 to 1.12)	**< 0.001**
CMT	0.01 (-0.02 to 0.04)	0.605		
Parafoveal NFL	-0.20 (-0.41 to 0.02)	0.092		
Parafoveal GCL	0.10 (-0.03 to 0.21)	0.067		
Parafoveal IPL	0.12 (-0.09 to 0.30)	0.213		

BCVA = best-corrected visual acuity; SE = spherical equivalent; IOP = intraocular pressure; DM = diabetes; CMT = central macular thickness; NFL = nerve fiber layer; GCL = ganglion cell layer, IPL = inner plexiform layer.

Values in boldface (P < 0.05) are statistically significant.

## Discussion

With the recent advent of noninvasive retinal imaging techniques such as OCT and OCTA, changes in the retinal structures and modifications of the macular capillary network have been evaluated in the DM patients with or without DR [5, 16]. In this study, we confirmed parafoveal inner retinal layer thinning, increased FAZ, and decreased VD of the SCP and DCP in T2DM patients without clinical DR using OCTA. In particular, there was more severe microvascular impairment of the DCP in patients with T2DM ≥ 10 years than in T2DM patients with relatively short disease duration or normal controls. Additionally, the BCVA was significantly associated with the VD in the SCP and DCP. Parafoveal GCL thickness was a significant factor associated with the VD of the SCP. With regard to the systemic factors in this study, hypertension and DM duration were significantly correlated with the VD of the DCP.

Previous studies have reported a reduction in the VD in T2DM patients without clinical DR. Cao et al. reported that the parafoveal and average VDs of the SCP and DCP decreased in patients without clinical DR compared to normal controls [9]. Zeng et al. also observed a decrease in the parafoveal and perifoveal VDs of the SCP and DCP in patients without clinical DR compared to the normal controls [17]. Similarly, in our study, we observed decreased superficial and deep VDs in T2DM patients, consistent with previous studies. Neuronal death and glial dysfunction following diabetic retinal neurodegeneration can cause a breakdown of the blood-retinal barrier (BRB), vasoregression, and impairment of neurovascular coupling, thereby, resulting in damage to the retinal neurovascular autoregulation. Such disruption of autoregulation may cause microvascular impairment reversely, which is a condition of positive feedback [18, 19].

However, we found that the superficial VD did not show a significant difference in the pairwise post hoc analyses. In a previous study, a prominent decrease in the superficial macular VD was reported in patients with prolonged T2DM than in patients with a relatively shorter duration of T2DM or normal controls by using 3 mm × 3 mm OCTA images according to the ETDRS subfoveal and parafoveal subfields [20]. This discrepancy may have occurred because different OCTA devices were used in the two studies. The age difference of the enrolled patients between the two studies would also cause a different result.

Unlike the superficial VD, a marked decrease in the deep VD was observed in patients with prolonged DM than in those with a relatively short period of DM. The more significant decreases in the VD in the deep retinal capillary layer might be attributed to the different anatomic structures of the deep and superficial layers. The density of the smaller vessels in the deep retinal capillary layer is greater than that in the superficial layer [21, 22]. We assumed that the deep capillary layer might be more vulnerable to hypoxia in diabetic retinal neurodegeneration because of higher vessel density of the smaller vessels. These findings would be also related to the development of microaneurysms that first appears in DCP by sluggish blood flow and microinfarction [23, 24]. These tendencies could become more pronounced with the accumulation of the damages caused by diabetic retinal neurodegeneration, thereby leading to more severely altered microvasculature in patients with prolonged T2DM.

Previous studies reported an association between the VD and visual acuity in DM patients [25, 26]. Meshi et al. found that superficial and deep capillary densities were negatively correlated with logMAR BCVA in DM patients without clinical DR [25]. Our study also found that both superficial and deep VD showed significant associations with the BCVA in T2DM patients without DR. In prolonged T2DM, compromise of the outer BRB could result in apoptosis of the photoreceptor layer and increased inflammation in the outer retina [27, 28]. Additionally, because of the impairment of choroidal circulation in T2DM, the inner retina may affect the blood supply of the outer retina, which is directly related to the visual function [29]. Therefore, altered superficial and deep microvasculatures could have a direct effect on the visual function in patients with T2DM despite lack of clinical signs of DR. Physicians should not overlook any alteration of the superficial and deep macular VD in T2DM patients regardless of any signs of clinical DR.

Toprak et al. reported a negative correlation of DM duration with neuroretinal alteration in patients with preclinical DR [30]. Sohn et al. found that the progressive retinal neurodegeneration was primarily related to the DM duration [31]. Our study indicated that the DM duration was a significant factor associated with the deep VD, and not with the superficial VD. The deep capillary layer might be more sensitive to neurovascular damage in T2DM, such as the disruption of the BRB and neurovascular unit impairment because of its higher density of smaller vessels than the superficial capillary layer. A previous study reported that in patients with T2DM, the age, age at diagnosis, and diabetes duration were independently associated with the macrovascular complications, but only diabetes duration was independently associated with microvascular complications, which is consistent with our study [32].

A previous study reported that the macular VD and perfusion density (PD) were significantly decreased and the inner retina was thinner in chronic hypertension (HTN) patients [33]. Sustained vasospasms of the retinal arterioles, reflect vasoconstriction as an autoregulatory response to HTN; this could cause compression of venules and lead to a decrease in the macular VD [34]. In our study, we found that factors including the DM duration and HTN were significantly associated with the deep VD and not the superficial VD. In patients with T2DM without clinical DR, the deep capillary layer might be more vulnerable to vasculopathy caused by HTN because it has more small vessels than the superficial capillary layer.

The signal strength is believed to be an important factor when analyzing the OCTA parameters in normal individuals and patients with various retinal diseases [35, 36]. Similarly, this study found that OCTA quality was significantly associated with the superficial and deep VD in T2DM patients. Because the OCTA parameters of the macular microvasculature are strongly influenced by the OCTA quality, physicians should be aware while analyzing OCTA parameters, that the comparison of the OCTA parameters would be inaccurate in the case of low signal strength.

A previous study reported that the average GCIPL thickness correlated significantly with the superficial macular VD [20]. We also found a significant association of the parafoveal GCL thickness with the superficial VD and not the deep VD. The superficial vascular complex supplies the ganglion cell complex formed by a combination of the nerve fiber layer (NFL), ganglion cell layer (GCL), and inner plexiform layer (IPL) [37]. We assumed that the GCL showed a significant association with the superficial VD because of these anatomical reasons.

Our study had several limitations. First, the retrospective nature of the study inevitably introduced some selection bias. Second, as the subjects with T2DM ≥ 10 years had a higher probability of having DR or other systemic diseases compared to other groups, the number of cases in the DM group 2 was relatively small, compared to other groups because of the strict inclusion criteria. Third, various information about visual functions, including the contrast sensitivity, color vision test, or microperimetry could not be obtained in our study. Fourth, we did not consider various systemic factors such as renal function and body mass index. The strengths of our study included OCTA images with OCTA quality greater than or equal to 20 for accurate analyses. Additionally, few studies have reported the impact of prolonged T2DM on the superficial and deep retinal microvasculatures in a relatively large number of cases.

In conclusion, patients with T2DM without clinical DR showed decreased VD of the SCP and DCP. In particular, there was more severe microvascular impairment of the DCP in patients with T2DM ≥ 10 years. Additionally, the superficial and deep VD were significantly associated with the BCVA. HTN and DM duration were significantly associated with the deep VD. Physicians should be aware that the diabetic retinal neurodegeneration and impairment of microvasculature, especially DCP, could persist over time; hence, a close and constant watch on the macular VD using OCTA is necessary.

## Supporting information

S1 Data (XLSX)
